# The interpretable CT-based vision transformer model for preoperative prediction of clear cell renal cell carcinoma SSIGN score and outcome

**DOI:** 10.1186/s13244-025-01972-0

**Published:** 2025-05-09

**Authors:** Kaiyue Zhi, Yanmei Wang, Lei Yan, Feng Hou, Jie Wu, Shuo Zhang, He Zhu, Lianzi Zhao, Ning Wang, Xia Zhao, Xianjun Li, Yicong Wang, Chengcheng Chen, Nan Wang, Yuchao Xu, Guangjie Yang, Pei Nie

**Affiliations:** 1 Department of Radiology, The Affiliated Hospital of Qingdao University, Qingdao, China; 2 GE Healthcare China, Pudong New Town, Shanghai, China; 3 Department of Nuclear Medicine, The Affiliated Hospital of Qingdao University, Qingdao, China; 4 Department of Pathology, The Affiliated Hospital of Qingdao University, Qingdao, China; 5 Department of Radiation Oncology, Fudan University Shanghai Cancer Center, Shanghai, China; 6 Department of Radiology, Shandong Provincial Hospital Affiliated to Shandong First Medical University, Jinan, China; 7 Department of Radiology, The Affiliated Hospital of Shandong University of Traditional Chinese Medicine, Jinan, China; 8 Department of Nuclear Medicine, Weifang People’s Hospital, Weifang, China; 9 Department of Medical Imaging, The Affiliated Hospital of Jining Medical College, Jining, China; 10 Department of Radiology, Rizhao People’s Hospital, Rizhao, China; 11 Department of Nuclear Medicine, Yantai Yuhuangding Hospital, The Affiliated Hospital of Qingdao University, Yantai, China; 12 School of Nuclear Science and Technology, University of South China, Hengyang City, China

**Keywords:** Vision transformer, Clear cell renal cell carcinoma, The stage, size, grade, and necrosis score, CT, Outcome

## Abstract

**Objectives:**

To develop and validate an interpretable CT-based vision transformer (ViT) model for preoperative prediction of the stage, size, grade, and necrosis (SSIGN) and outcome in clear cell renal cell carcinoma (ccRCC) patients.

**Methods:**

Eight hundred forty-five ccRCC patients from multiple centers were retrospectively enrolled. For each patient, 768 ViT features were extracted in the cortical medullary phase (CMP) and renal parenchymal phase (RPP) images, respectively. The CMP ViT model (CVM), RPP ViT model (RVM), and CMP-RPP combined ViT model (CRVM) were constructed to predict the SSIGN in ccRCC patients. The area under the receiver operating characteristic curve (AUC) was used to evaluate the performance of each model. Decision curve analysis (DCA) was used to evaluate the net clinical benefit. The endpoint was the progression-free survival (PFS). Kaplan–Meier survival analysis was used to assess the association between model-predicted SSIGN and PFS. The SHAP approach was applied to determine the prediction process of the CRVM.

**Results:**

The CVM, RVM, and CRVM demonstrated good performance in predicting SSIGN, with a high AUC of 0.859, 0.883, and 0.895, respectively, in the test cohort. DCA demonstrated the CRVM performed best in clinical net benefit. In predicting PFS, CRVM achieved a higher Harrell’s concordance index (*C*-index, 0.840) than the CVM (0.719) and RVM (0.773) in the test cohort. The SHAP helped us understand the impact of ViT features on CRVM’s SSIGN prediction from a global and individual perspective.

**Conclusion:**

The interpretable CT-based CRVM may serve as a non-invasive biomarker in predicting the SSIGN and outcome of ccRCC.

**Critical relevance statement:**

Our findings outline the potential of an interpretable CT-based ViT biomarker for predicting the SSIGN score and outcome of ccRCC, which might facilitate patient counseling and assist clinicians in therapy decision-making for individual cases.

**Key Points:**

- Current first-line imaging lacks preoperative prediction of the SSIGN score for ccRCC patients.
- The ViT model could predict the SSIGN score and outcome of ccRCC patients.
- This study can facilitate the development of personalized treatment for ccRCC patients.

**Graphical Abstract:**

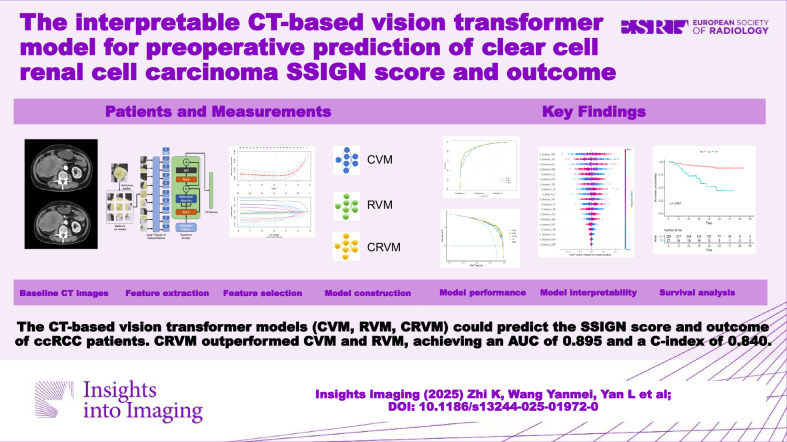

## Introduction

Renal cell carcinoma (RCC) is one of the most common malignant tumors in the urinary tract [1]. There are several subtypes of RCC, with clear cell renal cell carcinoma (ccRCC) being the most prevalent, accounting for approximately 70–80% of cases [2]. Although ccRCC is a disease that can be detected early and successfully treated with surgery or ablation methods, up to one-third of the cases may experience recurrence or metastasis [3], which makes the long-term survival rate for ccRCC patients not very optimistic. Therefore, preoperative risk stratification holds significant value for the personalized treatment approach of patients with ccRCC.

The stage, size, grade, and necrosis (SSIGN) score was a prognostic system developed by the Mayo Clinic in 2002 [4]. It was a multifactorial prognostic model that combined the tumor, node, metastasis (TNM) staging system, primary tumor size, nuclear grade, and coagulated tumor necrosis [5] to provide a simple tool for predicting tumor progression and prognosis in RCC patients, especially the ccRCC patients [6]. Several previous studies have found that, compared to other RCC prognostic models (including the TNM staging system, the UCLA integrated staging system [UISS]; the Leibovich score, and so on), the SSIGN score performed the best [7–9]. However, the SSIGN score is based on pathological parameters and relies on postoperative histologic status, which has several drawbacks, including difficulty in testing, delayed results, and limited predictive accuracy. Therefore, the development of a non-invasive and accurate predictive model for the SSIGN score is of great significance for clinical diagnosis and treatment.

Enhanced CT scans are a routine imaging examination for ccRCC patients, but they have limitations in obtaining tumor pathological grading information. Deep learning (DL) can process and analyze a large amount of medical imaging data, identify and quantify various tumor characteristics, thereby enabling quantitative analysis of tumor heterogeneity. In modern medical image analysis, DL techniques, particularly convolutional neural networks (CNNs), have been extensively applied to tasks of feature extraction and image classification. Currently, for renal tumors, DL has been utilized to assist in distinguishing between benign and malignant lesions [10], differentiating pathological subtypes [11], predicting pathological grading [11], and prognostic prediction [12]. Although CNNs are highly adept at feature extraction tasks, they cannot encode the relative positions of different features, which may result in the loss of global context for the features [13].

Transformer is a new type of neural network [14]. With the rise of the transformer architecture, a new paradigm—the vision transformer (ViT)—has begun to demonstrate powerful capabilities in the field of image recognition [13]. ViT leverages the self-attention mechanism to capture global context information [15], thereby achieving a comprehensive understanding of images [16]. In terms of disease classification and prognosis prediction, ViT has shown impressive performance in tasks such as the diagnosis of COVID-19 [17], the assessment of prognosis in colorectal cancer [16], the prediction of prostate cancer aggressiveness [18], and the prognosis evaluation of oropharyngeal cancer [19]. However, there is still a lack of research on preoperative prediction of the SSIGN score in ccRCC patients using ViT.

The purpose of this study was to develop and validate a CT-based ViT model for preoperative prediction of the SSIGN score and outcome in ccRCC patients using a multicenter dataset. We further employed SHAP (SHapley Additive exPlanations) to explore the interpretability of the ViT model.

## Methods

### Study design and patients

**Fig. 1:**
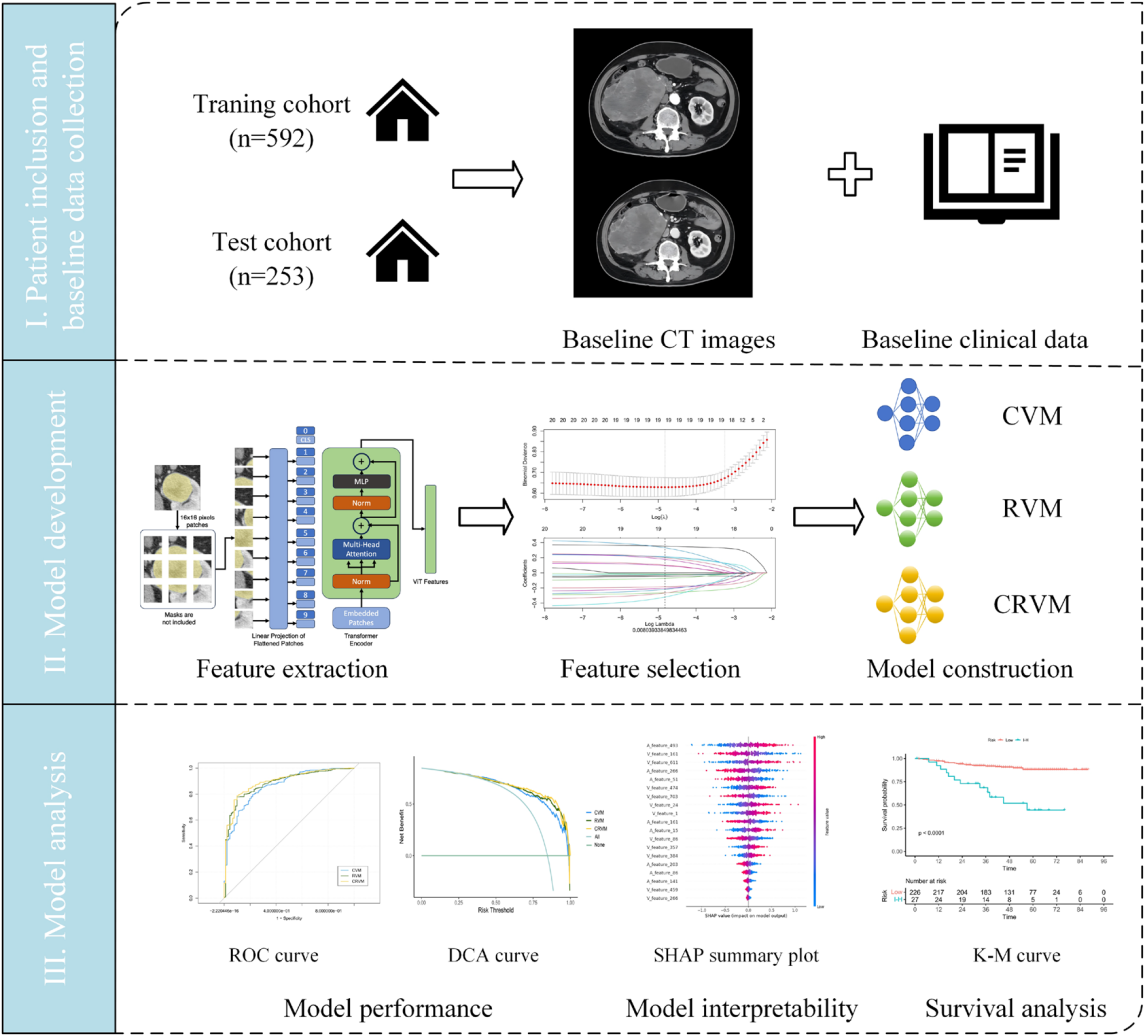
The design of the study. CVM, cortical medullary phase ViT model; RVM, renal parenchymal phase vision transformer model; CRVM, cortical medullary phase- renal parenchymal phase combined vision transformer model

This multicenter retrospective study was conducted in accordance with the Helsinki Declaration and approved by the institutional review board of participating hospitals, with a waiver of informed consent. The design of this study is shown in Fig. 1.

**Fig. 2:**
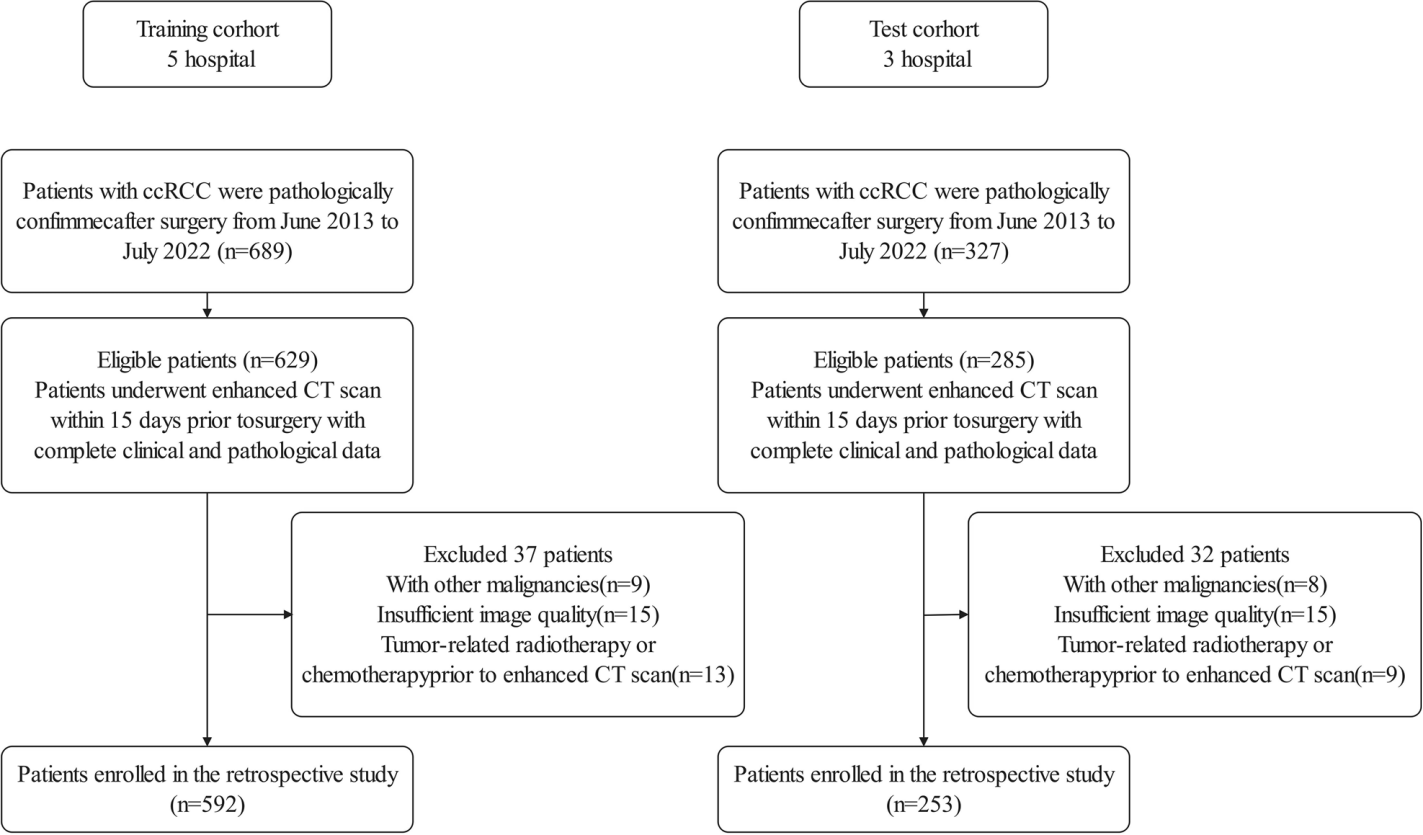
The patient recruitment pathway

Inclusion criteria: (1) pathologically confirmed ccRCC after surgery; (2) enhanced CT scan within 15 days prior to surgery with favorable image quality (overall image quality ≥ 4 [20]); (3) complete clinical and pathological data. Exclusion criteria: (1) tumor-related radiotherapy or chemotherapy prior to enhanced CT scan; (2) with other malignancies. Five hundred ninety-two patients from five hospitals constituted the training cohort, and 253 patients from 3 hospitals constituted the test cohort. The patient recruitment pathway is shown in Fig. 2, and the detailed recruitment pathway of patients from each center is shown in Fig. S1.

The baseline data, including age, gender, lumbago, Eastern Cooperative Oncology Group Performance Status (ECOG-PS), laboratory tests (alkaline phosphatase, lactate dehydrogenase, hemoglobin, leukocyte count, platelet count, hematuria, calcium, creatinine, and blood urea nitrogen), were collected.

### Pathological grading

All surgical specimens were stained with HE and reviewed by two pathologists with 8 years and 10 years of diagnostic experience, respectively. Any disagreement was resolved by consensus. The SSIGN score was calculated as 2 (pT1b) + 3 (pT2) + 4 (pT3a) + 4 (pT3b, pT3c, and pT4) + 2 (pN1 and pN2) + 1 (tumor size ≥ 10 cm) + 1 (grade 3) + 3 (grade 4) + 1 (necrosis), and 0 otherwise [21]. Patients were divided into two groups based on SSIGN score: low risk group (0–3) and intermediate-to-high risk group (≥ 4) [22].

### CT image acquisition

Patients were scanned in the supine position with breath-holding, ranging from the diaphragm to the lower edge of the kidney. Abdominal plain scan was performed first, and enhanced scans were performed twice at the cortical medullary phase (CMP, 30 s) and the renal parenchymal phase (RPP, 90 s) using a high-pressure injector to inject 90–100 mL of iodinated contrast medium (350 mg I/mL or 370 mg I/mL) at a flow rate of 2.5–3.0 mL/s. CT scanning parameters are shown in Table S1.

### Development and assessment of ViT models

Considering that the CT diagnosis of ccRCC typically requires the use of correlation information between different regions, we employ the ViT network as a feature extractor to better represent the global dependencies within CT images. The CT scans were configured to the soft tissue window settings (width of 350, level at 50), and then translated into a grayscale palette ranging from 0 to 255. We used the ITK-SNAP software (version 3.8, www.itksnap.org) to achieve three-dimensional (3D) segmentation of the region of interest (ROI). The contours were drawn along the tumor margins on CMP and NP images, while avoiding covering the adjacent renal parenchyma and perirenal fat. The largest cross-section was derived from the three-dimensional region of interest, and was extended outward into a square area. The cropped area was resized to 224 × 224 pixels and duplicated into three channels as the GRB. It was then divided into patches of 16 × 16 pixels. Further, the data was normalized to the [0, 1] range. After this preprocessing, differences introduced by multicenter images were standardized prior to feature extraction. ViT features were extracted from the largest cross-section by the pre-trained classification model, Google/vit-base-patch16-224. For each patient, 768 ViT features were extracted in the CMP and the RPP images, respectively.

By utilizing minimum redundancy maximum relevance (mRMR) and least absolute shrinkage and selection operator (LASSO) for dimensionality reduction of CMP ViT features, RPP ViT features, the CMP-RPP combined ViT features, we established the Cortical medullary phase vision transformer model (CVM), the RPP ViT feature model (RVM), as well as the CMP-RPP combined feature ViT model (CRVM) by multiple logistic regression analysis.

The area under the receiver operating characteristic ROC curve (AUC), accuracy, sensitivity, and specificity were used to evaluate the predictive performance of the models. The comparison of AUCs of the CVM, RVM, and CRVM was analyzed by the DeLong test. Decision curve analysis (DCA) was used to evaluate the net clinical benefit of each model in predicting the SSIGN score for ccRCC.

### The interpretability of the models

To enhance the transparency and credibility of the model, we employed the SHAP method to improve the interpretability of our machine learning models [23]. SHAP is a game-theoretic approach that provides a unified measure of feature importance, ensuring a fair assessment of each feature’s contribution to the model’s output [24]. And it can be used to explain how each feature contributes to increasing or decreasing the probability of individual outcomes [25].

### Follow-up and survival analysis

Patients were followed up every 6–12 months for the first two years after surgery, and then annual follow-up was conducted. Follow-up information was derived from medical records, including physical examination results, tumor markers, CT or MRI imaging results, or telephone follow-up. The final follow-up date was July 30, 2022. The endpoint was progression-free survival (PFS), defined as the time from the start of treatment or from the time of diagnosis until there is evidence of disease progression.

The correlation between the model-predicted SSIGN and PFS was evaluated using Kaplan–Meier survival analysis. The log-rank test was used to assess survival differences between low-risk and intermediate-to-high-risk groups. Harrell’s concordance index (*C*-index) and hazard ratio (HR) were used to assess the performance of these models.

### Statistics

The mRMR, LASSO regression analysis, multiple logistic regression analysis, Delong test, ROC analysis, DCA, SHAP, and survival analysis were performed with R statistical software (Version 3.3.3, https://www.r-project.org). *p* < 0.05 was considered statistically significant.

## Results

### Clinical features

A total of 845 patients were enrolled, with a median age of 57 years. Among these 845 patients, there were 558 males and 287 females. The detailed clinical features of the patients in both the training and test cohorts were presented in Table 1.

**Table 1:** Clinical features of the patients with ccRCC

Variable	Training (*n* = 592)	Test (*n* = 253)	*p*-value
Age $(\overline{x}\pm {{{\rm{SD}}}})$, year	56.20 ± 10.43	56.00 ± 11.26	0.80
Gender (male/female)	398/194	160/93	0.26
Hematuria (absent /present)	510/82	220/33	0.75
Lumbago (absent /present)	475/117	204/49	0.89
ECOG_PS (0/1/2)	325/260/7	143/109/1	0.54
Hemoglobin (normal/abnormal)	476/116	196/57	0.33
Leukocyte count (≤ 10 × 10^9^/L/> 10 × 10^9^/L)	548/44	237/16	0.57
Platelet count (≤ 320 × 10^9^/L/> 320 × 10^9^/L)	541/51	228/25	0.56
Lactic dehydrogenase (≤ 367.5 U/L/> 367.5 U/L)	510/82	215/38	0.66
Alkaline phosphatase $(\overline{x}\pm {{{\rm{SD}}}})$, U/L	79.85 ± 40.12	81.11 ± 61.02	0.72
Calcium (≤ 2.75 mmol/L/> 2.75 mmol/L)	584/8	249/4	0.80
Creatinine $(\overline{x}\pm {{{\rm{SD}}}})$, umol/L	72.83 ± 21.23	72.22 ± 19.31	0.69
Blood urea nitrogen $(\overline{x}\pm {{{\rm{SD}}}})$, mmol/L	5.86 ± 5.57	5.60 ± 3.71	0.50

### CVM, RVM, and CRVM development and validation

We used two feature selection methods. In the first step, mRMR was performed to eliminate redundant and irrelevant features, 30 features were retained. Then, LASSO was conducted to choose the optimized subset of features to construct the final model. Finally, 17 CMP ViT features, 16 RPP ViT features, and 19 CMP-RPP combined ViT features were selected to develop the CVM, RVM, and CRVM, respectively. The details were presented in Fig. S2.

**Fig. 3:**
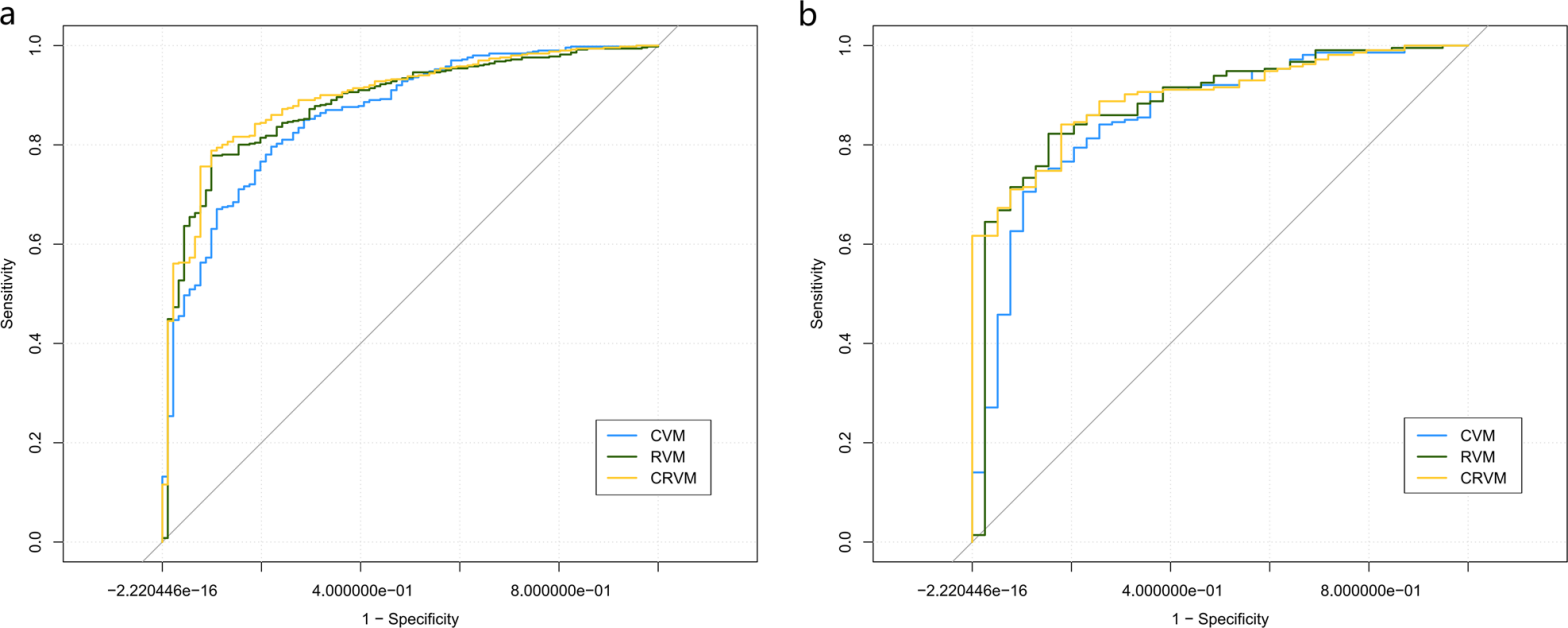
The ROC curves of the CMP ViT feature model (CVM), RPP ViT feature model (RVM), and CMP-RPP combined feature ViT model (CRVM) (**a** training cohort, **b** test cohort) for predicting the stage, size, grade, and necrosis (SSIGN) score in patients with ccRCC

**Fig. 4:**
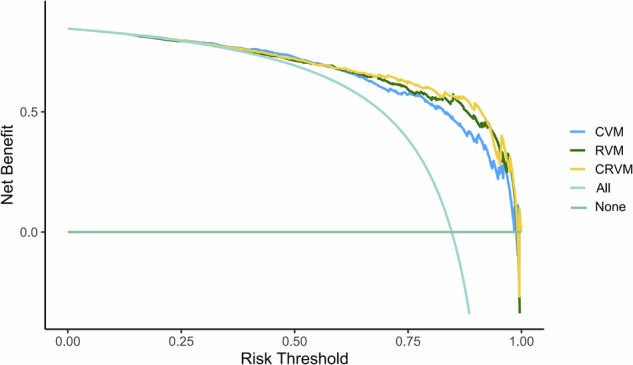
The DCA of the CMP ViT feature model (CVM), RPP ViT feature model (RVM), and CMP-RPP combined feature ViT model (CRVM)

The performance of the CVM, RVM, and CRVM in predicting the SSIGN score of the ccRCC patients is shown in Table 2 and Fig. 3. The Delong test demonstrated statistically significant differences in predictive efficiency between the CVM and CRVM (*p* = 0.027) in the training cohort. DCA showed that the CRVM had a higher clinical net benefit than the CVM or RVM (Fig. 4).

**Table 2:** The performance of the ViT models in predicting the SSIGN score of patients with ccRCC

Models	Cohort	AUC (95%CI)	Accuracy	Sensitivity	Specificity
CVM	Training cohort	0.863 (0.833–0.890)	0.794	0.796	0.789
	Test cohort	0.859 (0.810–0.900)	0.767	0.748	0.872
RVM	Training cohort	0.885 (0.856–0.909)	0.797	0.778	0.901
	Test cohort	0.883 (0.837–0.920)	0.826	0.822	0.846
CRVM	Training cohort	0.895 (0.868–0.919)	0.806	0.788	0.901
	Test cohort	0.895 (0.851–0.930)	0.838	0.841	0.821

*CVM* CMP vision transformer feature model, *RVM* RPP vision transformer feature model, *CRVM* CMP-RPP combined feature vision transformer model, *AUC* the area under the receiver operating characteristic curve, *CI* confidence interval

### The interpretability of the models

**Fig. 5:**
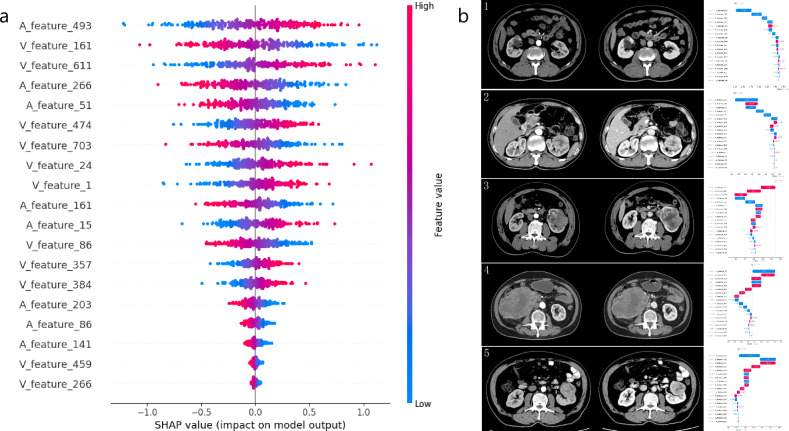
SHAP summary plot of the CRVM (**a**) depicts the features of global prediction impact on the decision and interaction between features. The importance of features was listed top-down. Each point represents the SHAP value of a patient feature. Dots to the left of the *Y*-axis increase the chances of having a lower SSIGN score, while dots to the right increase the chances of having a higher SSIGN score. The SHAP waterfall plots (**b**) showed the individual interpretability of CRVM. From left to right, the CMP CT images, the RPP CT images, and the corresponding SHAP waterfall plots for each patient were displayed. Patients (**b** [1], **b** [2]) exhibited final predicted values that were below the base value, leading the CRVM to classify them as low risk for SSIGN. Patients (**b** [3], **b** [4], **b** [5]) had final predicted values exceeding the base value, resulting in a CRVM classification of intermediate-to-high risk for SSIGN. Pathology confirmed these risk assessments in patients (**b**) (1, 2, 3, 4), while the risk assessment in patient (**b**) (5) was inconsistent with the pathological results

As shown in Fig. 5a, the SHAP summary plot of the CRVM shows the effect of each feature on the prediction model. In the SHAP summary plot of the CRVM, each point corresponds to an individual SHAP value for a feature across patients, utilizing a color scale that transitions from red for the highest to blue for the lowest feature values. SHAP dependence plots were created to explain the influence of certain features on the model prediction. A positive SHAP value indicates a higher SSIGN score for each prediction and vice versa for a negative value. The SHAP waterfall plots of 5 representative ccRCC patients are shown in Fig. 5b. The SHAP waterfall plot of the CRVM showed the individual interpretability. Red bars indicate increased predictive value, and blue bars indicate decreased predictive value. Under the influence of all features, a final predictive value is obtained, and if this value is less than the base value, the patient is predicted to have a low SSIGN score.

### Survival prediction

As of the last follow-up, the recurrence rate was 15.1% (128/845), and the median PFS of the SSIGN low-risk, and SSIGN intermediate-to-high risk patients was 53 months (range, 1–118 months), and 36.5 months (range, 1–85 months), respectively.

**Fig. 6:**
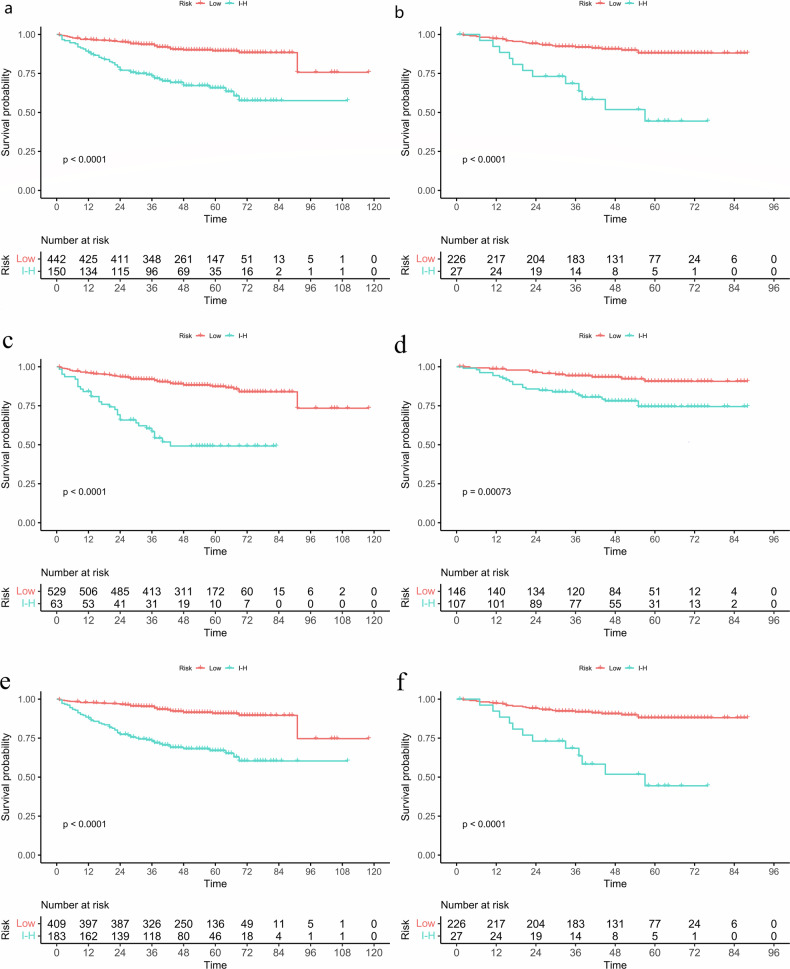
Kaplan–Meier survival curves for PFS by the CMP ViT feature model (CVM) (**a** training cohort, **b** test cohort), RPP ViT feature model (RVM) (**c** training cohort, **d** test cohort), and CMP-RPP combined feature ViT model (CRVM) (**e** training cohort, **f** test cohort) in patients with ccRCC. Low, SSIGN low risk; I-H, SSIGN intermediate-to-high risk

The Kaplan–Meier survival curves for PFS in low-risk and intermediate-to-high-risk SSIGN patients stratified by the CVM, RVM, and CRVM are shown in Fig. 6. There was a significant association between low-risk and intermediate-to-high-risk SSIGN patients identified by these models (log-rank test, *p* < 0.05). *C*-index and HR estimates for the CVM, RVM, and CRVM in predicting the PFS of ccRCC patients are shown in Table 3 and Table S2, respectively.

**Table 3:** The performance of the ViT models in predicting PFS of the patients with ccRCC

*C*-index (95% CI)	Training cohort	Test cohort
CVM	0.804 (0.737–0.871)	0.719 (0.560–0.878)
RVM	0.849 (0.792–0.907)	0.773 (0.645–0.901)
CRVM	0.835 (0.774–0.897)	0.840 (0.748–0.932)

*CVM* CMP vision transformer feature model, *RVM* RPP vision transformer feature model, *CRVM* CMP-RPP combined feature vision transformer model, *CI* confidence interval

## Discussion

The purpose of this multicenter study was to develop and validate an interpretable CT-based ViT model in predicting the SSIGN score and outcome in patients with ccRCC. We found the CRVM provided favorable predictive value for the SSIGN score. Further, SHAP analysis visualized how ViT features influenced the prediction of the SSIGN score, thereby improving the interpretability of the CRVM as a reliable tool for assessing the SSIGN score. In addition, the CRVM predicted SSIGN risk group was associated with PFS in patients with ccRCC.

The SSIGN score stands as a widely utilized prognostic tool for patients with ccRCC. Integrating factors such as tumor size, lymph node status, distant metastasis, and tumor grade, it provides a quantifiable method for assessing patient prognosis [6]. Preoperative prediction of the SSIGN score is instrumental in formulating personalized treatment strategies, potentially enhancing patient outcomes by optimizing surgical approaches and minimizing unnecessary treatments.

Previous studies have indicated that adverse CT features such as primary tumor size, ill-defined margins, and presence of infiltration may be correlated with an increased aggressiveness of ccRCC [26]. While CT plays a pivotal role in the diagnosis of ccRCC, offering insights into tumor size and morphology, it falls short in predicting pathological states such as tumor grading and lymph node metastasis. The resolution and contrast of CT images may not suffice to reveal the micro-features of the tumor, thereby limiting their utility in preoperative prediction of SSIGN scores.

Artificial Intelligence (AI), encompassing radiomics and DL, has demonstrated significant potential in non-invasively characterizing tumor heterogeneity [27]. The radiomics and DL played a crucial role in identifying the pathological and biological behaviors of ccRCC, and have been instrumental in predicting the risk stratification, including the prediction of the TNM stage [28], Fuhrman grade [29, 30], WHO/ISUP classification [31], Leibovich score [32], and nuclear grade [33] for patients with ccRCC. At present, there are relatively few studies on predicting the SSIGN score of ccRCC patients using radiomics or DL. Choi et al analyzed T2-weighted and T1-contrast-enhanced MRI images of 364 ccRCC patients, and established a radiomics nomogram for predicting SSIGN scores. They found that this model could accurately stratify ccRCC patients into the SSIGN low-risk group and the SSIGN high-risk group, achieving an AUC of 0.89 in the test cohort [34]. Jiang et al analyzed the CT images of 330 patients with ccRCC and established a radiomic signature capable of predicting low-risk and intermediate to high-risk SSIGN scores (AUC: 0.876–0.928 in the validation cohorts) [22]. A study [12] has analyzed the CT images of 784 ccRCC patients and established a three-class DL radiomics model, which classified ccRCC patients into low-, intermediate-, and high-SSIGN score groups (*C*-index: 0.824–0.827 in the test cohorts).

ViT is an emerging DL architecture that captures global contextual information in images through self-attention mechanisms. Compared to CNNs, ViT exhibits stronger global perception, higher computational efficiency, and enhanced generalization capabilities [35]. A study enrolled 1729 colonoscopy images from patients undergoing surgery for colorectal cancer (CRC) and developed a model based on a feature ensemble vision transformer (FEViT), revealing that the FEViT (AUC = 0.93) outperformed the TNM staging (AUC = 0.83) in predicting the prognosis of CRC patients [16]. Chen et al [36] analyzed 786 CT images of bone tumors and established a fusion model, VGG16-ViT, by leveraging the advantages of the VGG-16 network and the ViT model, discovering that the VGG16-ViT possesses robust classification capabilities for bone tumor datasets (AUC = 0.97). However, there is still a lack of research on preoperative prediction of SSIGN scores in ccRCC patients using ViT. In this study, we utilized a multicenter dataset to develop and validate an interpretable CT-based ViT model, for predicting the SSIGN score of ccRCC patients. The results indicated that the CRVM demonstrated strong predictive performance, with AUC scores of 0.895 in both the test and training cohorts. CRVM demonstrated greater robustness in predicting the SSIGN score of ccRCC patients.

The SHAP method was used to assign contribution values to each feature for the model predictions, visualizing the extent to which the down-scaled features contributed to the assessment of the SSIGN score. This confirmed the significant potential of the ViT features in predicting the SSIGN score, allowing doctors to better understand the model’s decision-making process. In our study, the summary plot of the CRVM was used to show the effect of each feature on the prediction model, and SHAP waterfall plots were used to demonstrate CRVM predictive SSIGN outcomes in 5 representative ccRCC patients. The accuracy of CRVM in the training and the test cohort was 0.806 and 0.838, respectively. The pre-trained model was developed by Google (https://github.com/google-research/vision_transformer) for realistic photo classification tasks. It does not learn from SSIGN scores. AI features may not be perfect for many corner cases, as shown in case 5 in Fig. 5b. In the future, minor adjustments to the features may give promising diagnostic results for these corner cases.

Previous studies have demonstrated the predictive value of radiomics and DL in risk stratification in patients with ccRCC. Yang et al [37] found that the radiomics model that combined clinical and CT radiomics features could be used to predict recurrence-free survival in localized ccRCC patients with a *C*-index of 0.704 in the test cohort. Chen et al [38] found the DL model could be utilized to predict the disease-free survival status of ccRCC patients with an AUC of 0.917 and 0.900 in the test cohort. In our study, beyond predicting SSIGN score, we further analyzed the prognostic value of the CRVM. Utilizing the SSIGN score as a stratification factor, we assessed the performance of the CRVM in predicting the prognosis of ccRCC patients. There was a significant difference in PFS between the low-risk and intermediate-to-high risk groups predicted by the CRVM, and the CRVM achieved a higher *C*-index than the CVM and RVM did in the test cohort, indicating the prognostic value of the CT-based CRVM in the management of ccRCC patients. For patients identified as low metastatic risk by the CRVM, postoperative management typically includes standard follow-up and treatment protocols. In contrast, patients with intermediate-to-high metastatic risk are advised to undergo aggressive adjuvant chemotherapy and radiotherapy after surgery. Additionally, they may consider targeted therapy or immunotherapy regimens as appropriate.

There are several limitations. Firstly, this study was retrospective, which may hamper its reproducibility and generalization. For example, the CT scans were acquired with different scanners with different characteristics, and this might affect the Vit features. Therefore, we performed normalization preprocessing on these images, and then differences introduced by multicenter images were standardized prior to feature extraction. Further prospective studies are needed to further validate the effectiveness of the model. Secondly, we primarily focused on the capability of the ViT model and did not fully consider the features of traditional CNNs and their potential complementary nature with ViT features. In addition, clinical and CT features were not analyzed. Thus, future studies will integrate the features of CNNs and ViT to construct a more comprehensive feature representation, thereby enhancing the model’s performance and robustness in complex visual tasks. In addition, the construction of clinical and CT feature models and the comparison of their efficiency will be included in the future.

## Conclusion

In conclusion, the interpretable CT-based CRVM may serve as a non-invasive biomarker in predicting the SSIGN score and outcome of ccRCC, thus guiding individualized management of ccRCC patients and promoting precise medicine.

## Supplementary information

ELECTRONIC SUPPLEMENTARY MATERIAL

## Data Availability

The datasets used and/or analyzed during the current study are available from the corresponding author on reasonable request.

## Abbreviations

AUC: Area under the receiver operating characteristic curve
ccRCC: Clear cell renal cell carcinoma
CI: Confidence interval
*C*-index: Harrell’s concordance index
CMP: Cortical medullary phase
CNNs: Convolutional neural networks
CRVM: Cortical medullary phase-renal parenchymal phase combined vision transformer model
CVM: Cortical medullary phase vision transformer model
DCA: Decision curve analysis
DL: Deep learning
ECOG-PS: Eastern Cooperative Oncology Group Performance Status
HR: Hazard ratio
LASSO: Least absolute shrinkage and selection operator
mRMR: Minimum redundancy maximum relevance
OR: Odds ratio
PFS: Progression-free survival
RCC: Renal cell carcinoma
RPP: Renal parenchymal phase
RVM: Renal parenchymal phase vision transformer model
SHAP: Shapley additive explanations
SSIGN: Stage, size, grade, and necrosis
TNM: Tumor, node, metastasis
ViT: Vision transformer
