# Supplementary material for: The interpretable CT-based vision transformer model for preoperative prediction of clear cell renal cell carcinoma SSIGN score and outcome
Source: Insights Imaging. 2025 May 9;16:98. doi: 10.1186/s13244-025-01972-0 (PMC12064486; doi:10.1186/s13244-025-01972-0)
Supplement: Supplementary file 1 — ELECTRONIC SUPPLEMENTARY MATERIAL [file 13244_2025_1972_MOESM1_ESM.pdf]

The interpretable CT-based vision transformer model for preoperative prediction of clear cell renal cell carcinoma SSIGN score and outcome

ELECTRONIC SUPPLEMENTARY MATERIAL

Insights Imaging (2025) Zhi K, Wang Yanmei, Yan L, et al.

Table S1 CT scan parameters

CT scanner	CT 256	CT 256	CT 128	CT 128	CT 64	CT 64	CT 64	CT 16	CT 16
Scanner model	Brilliance iCT 256	Revolution	Somatom Definition	Somatom Definition Flash	LightSpeed VCT	Somatom Sensation 64	Discovery 750	Brilliance 16	Brightspeed 16
Manufacturer	Philips	General Electric	Siemens	Siemens	General Electric	Siemens	General Electric	Philips	General Electric
Gantry rotation time (s)	0.5	0.28	0.33	0.28	0.5	0.5	0.5	0.5	0.6
Tube voltage (kV)	120	120	120-	120	120	120	120	120	120
Tube current	250 mA	250-400 mA (automatic tube current modulation)	200 mAs	Ref. 200 mAs (Care Dose 4D)	200 mA	200 mAs	200-400 mA (automatic tube current modulation)	200 mAs	100-400 mA
Detector collimation (mm)	0.625	0.625	0.6	0.6	0.625	0.6	0.625	0.75	0.625
Matrix	512×512	512×512	512×512	512×512	512×512	512×512	512×512	512×512	512×512
Pitch	0.915	0.992	1.0	1.0	0.984	1.0	1.375	1	1.375

Slice thickness (mm)	5	5	5	5	5	5	5	5	5
Hospital	a, c	a, f	b, d	a, e, h	c	a, b	g	a	g

Note- s = second, kV = kilovolt, mA = milliampere, mm = milimetre. a, The Affiliated Hospital of Qingdao University; b, Fudan University Shanghai Cancer Center; c, Yantai Yuhuangding Hospital; d, Weifang People's Hospital; e, The Affiliated Hospital of Jining Medical College; f, The Affiliated Hospital of Shandong University of Traditional Chinese Medicine; g, Shandong Provincial Hospital Affiliated to Shandong First Medical University; h, Rizhao People's Hospital.

Table S2 The hazard ratio (HR) estimates associated with progression-free survival stratified by ViT models in the clear cell renal cell carcinoma

Model	Subgroup	Training cohort		Test cohort	
		HR	95% CI	HR	95% CI
CVM	Intermediate-to-high risk vs. low risk	3.991	2.454-6.490	2.589	0.869-7.709
RVM	Intermediate-to-high risk vs. low risk	5.123	2.435-10.778	3.195	1.627-6.272
CRVM	Intermediate-to-high risk vs. low risk	4.548	2.887-7.165	5.508	1.695-17.897

Note- CVM= cortical medullary phase (CMP) vision transformer feature model; RVM= renal parenchymal phase (RPP) vision transformer feature model; CRVM= CMP-RPP combined feature vision transformer model; CI= confidence interval.

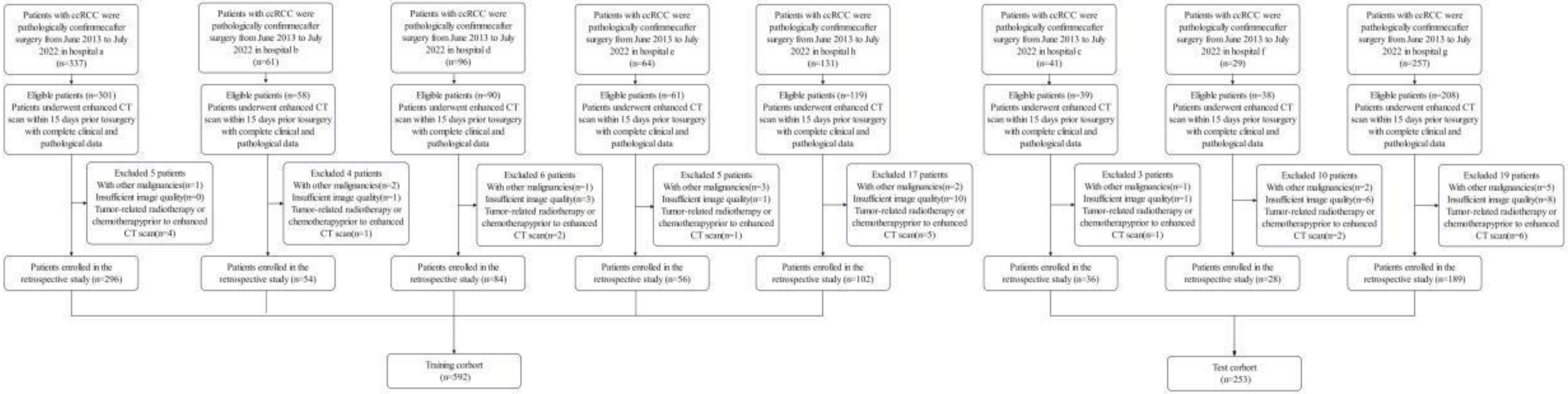

Figure S1 The detailed recruitment pathway of patients from each center.

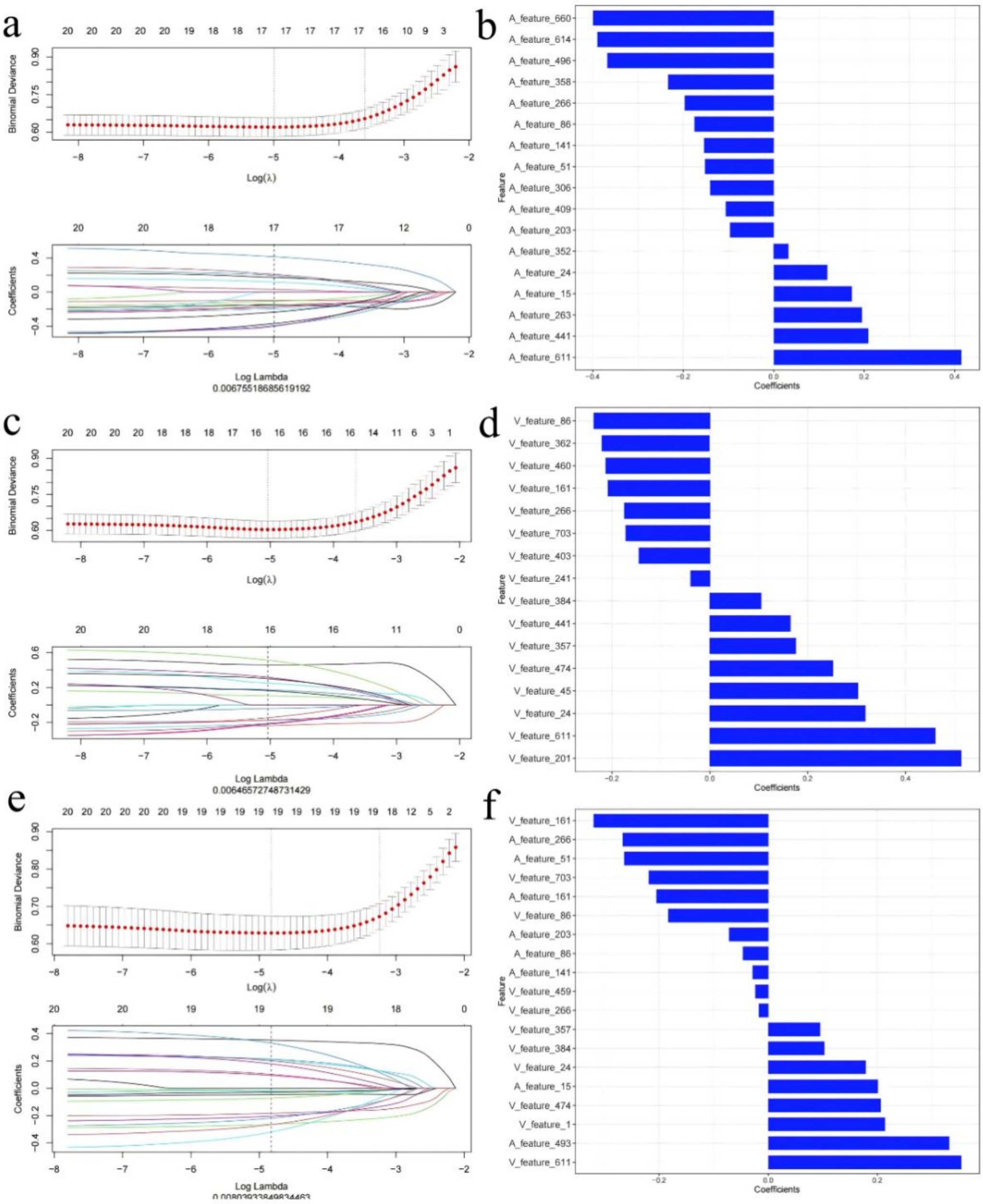

Figure S2 The ViT features selection by using the LASSO regression model (a, cross-validation parameter selection process and the trajectory dynamics of each cortical medullary phase [CMP] features; b, the final selected CMP features; c, cross-validation parameter selection process and the trajectory dynamics of each renal parenchymal phase [RPP] features; d, the final selected RPP features; e, cross-validation parameter selection process and the trajectory dynamics of each CMP-RPP combined features; f, the final selected CMP-RPP combined features).

Checklist for Artificial Intelligence in Medical Imaging (CLAIM): 2024 Update

Section / Topic	No.	Item	Page / Line	No	NA
TITLE / ABSTRACT					
	1	Identification as a study of AI methodology, specifying the category of technology used (e.g., deep learning)	1/1		
ABSTRACT					
	2	Summary of study design, methods, results, and conclusions	1/5		
INTRODUCTION					
	3	Scientific and/or clinical background, including the intended use and role of the AI approach	4/8		
	4	Study aims, objectives, and hypotheses	6/9		
METHODS					
Study Design	5	Prospective or retrospective study	6/16		
	6	Study goal	6/18		
Data	7	Data sources	7/2		
	8	Inclusion and exclusion criteria	6/20		
	9	Data pre-processing	8/15		
	10	Selection of data subsets	8/12		
	11	De-identification methods	8/7		
	12	How missing data were handled			√
	13	Image acquisition protocol	7/19		
Reference Standard	14	Definition of method(s) used to obtain reference standard	7/11		
	15	Rationale for choosing the reference standard			√
	16	Source of reference standard annotations			√
	17	Annotation of test set			√
	18	Measures of inter- and intra-rater variability of features described by the annotators	7/12		

Data Partitions	**19**	How data were assigned to partitions	**7/3**		
	**20**	Level at which partitions are disjoint	**7/5**		
Testing Data	**21**	Intended sample size			√

Section / Topic	No.	Item	Page / Line	No	NA
Model	**22**	Detailed description of model	**8/3**		
	**23**	Software libraries, frameworks, and packages	**8/10**		
	**24**	Initialization of model parameters	**8/18**		
Training	**25**	Details of training approach			√
	**26**	Method of selecting the final model			√
	**27**	Ensembling techniques			√
Evaluation	**28**	Metrics of model performance	**9/5**		
	**29**	Statistical measures of significance and uncertainty	**10/11**		
	**30**	Robustness or sensitivity analysis	**9/6**		
	**31**	Methods for explainability or interpretability	**9/11**		
	**32**	Evaluation on internal data	**9/7**		
	**33**	Testing on external data	**9/7**		
	**34**	Clinical trial registration			√
**RESULTS**					
Data	**35**	Numbers of patients or examinations included and excluded	**10/16**		
	**36**	Demographic and clinical characteristics of cases in each partition	**10/16**		
Model performance	**37**	Performance metrics and measures of statistical uncertainty	**11/4**		
	**38**	Estimates of diagnostic performance and their precision	**11/4**		
	**39**	Failure analysis of incorrect results	**15/11**		
**DISCUSSION**					
	**40**	Study limitations	**16/12**		
	**41**	Implications for practice, including intended use and/or clinical role	**16/3**		
**OTHER INFORMATION**					

	**42**	Provide a reference to the full study protocol or to additional technical details	**11/3**		
	**43**	Statement about the availability of software, trained model, and/or data	**17/20**		
	**44**	Sources of funding and other support; role of funders	**18/3**		

* Indicate page and/or line number for each checklist item that is present. NA = not applicable.
